# Alterations of the Intestinal Barrier and Inflammatory Response, Caused by Chronic Ozone Exposure in a Rat Model

**DOI:** 10.3390/antiox14081000

**Published:** 2025-08-15

**Authors:** Alfredo Miranda-Martínez, Erika Rodríguez-Martínez, Marlen Valdés-Fuentes, Selva Rivas-Arancibia

**Affiliations:** Departamento de Fisiología, Facultad de Medicina, Universidad Nacional Autónoma de México, Mexico City 04510, Mexico; fisiomedicina@gmail.com (A.M.-M.); arodriguez@facmed.unam.mx (E.R.-M.); marlen_valdes@ciencias.unam.mx (M.V.-F.)

**Keywords:** ozone exposure, oxidative stress, intestinal barrier, inflammation, intestinal permeability

## Abstract

Ozone pollution is a significant public health problem due to its association with chronic diseases. This study examines the effects of repeated exposure to low doses of ozone on intestinal barrier function in rats. Seventy-two male Wistar rats were divided into six groups. The control group was exposed to normal air, while the ozone groups received a dose of 0.25 ppm for four hours daily for periods of 7, 15, 30, 60, and 90 days, respectively. After treatment, the duodenum, jejunum, and colon were removed and analyzed by biochemical assays, Western blot, immunohistochemistry, and histological techniques. The results indicated an increase in oxidized lipids and structural alterations in the duodenum and jejunum after 7 days of ozone exposure. The result showed changes in haptoglobin, IL-1β, and IL-6. In addition, increased immunoreactivity varied according to intestinal structure and the duration of ozone exposure in the duodenum, jejunum, and colon. In conclusion: Ozone exposure causes an increase in proinflammatory cytokines that leads to a loss of regulation of the immune response in the duodenum, jejunum, and colon of rats, as well as structural changes that alter the intestinal barrier and perpetuate a state of chronic inflammation characteristic of inflammatory bowel diseases.

## 1. Introduction

Air pollution is a public health concern, especially in highly industrialized and urbanized regions [1,2,3]. Ozone (O_3_) is one of the most abundant and highly reactive pollutants. Upon entering the body, it generates reactive oxygen species (ROSs) that oxidize and damage macromolecules, including lipids, proteins, and nucleic acids [4,5]. The increase in ROSs produced secondary to repeated exposure to environmental ozone pollution causes alterations at multiple levels, both molecular and in organs and systems, and ultimately induces degenerative processes in the body. Physiologically, ROSs are produced as part of cellular metabolism, mainly by mitochondria, the endoplasmic reticulum, and peroxisomes [6]. ROSs are also important signalers of cell differentiation and proliferation mechanisms, migration, angiogenesis, and modulators of the immune system in the defense against inflammatory components and responses [7,8].

Immune system cells recognize oxidized molecules, particularly lipids, as pathogens through Toll-like receptors (TLRs) and initiate cell signaling pathways that activate the synthesis and release of inflammatory mediators [9,10]. Inflammatory responses involve complex signaling pathways where interactions between pro-inflammatory and anti-inflammatory mediators regulate the immune response. During the oxidation–reduction balance, an inflammatory response is established during which pro- and anti-inflammatory cytokines act in a finely coordinated response to enable successful recovery from injuries, trauma, sepsis, and infections, aiding in the removal of harmful stimuli and the initiation of healing processes, contributing to the restoration of tissue homeostasis [11,12]. Therefore, the inflammatory response is reparative and self-limiting.

Chronic inflammation occurs when the body is unable to regulate inflammatory processes over time [13]. Research indicates that a loss of redox balance contributes to the dysregulation of inflammatory responses and plays a significant role in the development of various health issues, including neurodegenerative diseases [14,15,16,17], cardiovascular diseases [18], endothelial dysfunction [19], cancer, metabolic syndrome [20], obesity [21], and COVID-19 [22].

In animal models, chronic and repeated exposure to low doses of O_3_ has been shown to increase oxidative molecules and weaken antioxidant systems, leading to a sustained inflammatory response that intensifies with continued exposure [23]. Additionally, this exposure causes mitochondrial and endoplasmic reticulum swelling, decreases ATP production in mitochondria, alters the structural conformation of proteins, and impairs tissue repair capacity [23,24,25].

In the gastrointestinal tract, various ROSs are produced as antimicrobial agents and redox signaling molecules. These ROSs are generated by epithelial cells, endothelial cells, and innate immune cells to protect the intestinal epithelium [26]. The intestine plays a crucial role in coordinating digestion, absorption, secretion, and the activities of the microbiota, immune system, endocrine system, and peripheral nervous system. These functions can be influenced by diet, exercise, medication, chronic oxidative stress, and factors such as exposure to environmental pollutants, among others [27,28]. The functions that the gut performs though its constant exposure to food, microbiota, and pathogens indicated that it has a highly specialized immune system associated with it. This system ranges from maintaining tissue integrity and repairing tissue injuries to absorbing food and water and eliminating pathogenic invaders [29,30].

Under normal physiological conditions, the intestine produces various cytokines, hormones, neurotransmitters, and enzymes essential for its proper functioning [31]. However, when the intestinal barrier loses its selective permeability, large molecules can enter the bloodstream. The immune system perceives these substances as threats, triggering inflammatory responses. When these responses become chronically unregulated, they are associated with inflammatory intestinal disorders and extraintestinal autoimmune diseases, such as rheumatoid arthritis and multiple sclerosis, as well as metabolic conditions like diabetes and obesity [32]. We are interested in studying the effects of repeated exposure to low doses of O_3_ on the intestinal barrier and the changes in the inflammatory interleukin-1 beta (IL-1β) and interleukin-6 (IL-6) in the duodenum, jejunum, and colon of rats.

## 2. Materials and Methods

The experiments conducted in this study strictly adhered to the guidelines set forth by the Mexican Official Standard NOM-062-ZOO-1999 [33], which specifies the technical requirements for the production, care, and use of laboratory animals. International guidelines on animal ethics and management were also followed to minimize both the number of animals used and their suffering. The Ethics Committee of the UNAM Faculty of Medicine approved all animal experiments [34]. A total of 72 male Wistar rats, weighing 250 g, were individually housed in transparent acrylic cages with laboratory animal chow (LabDiet, México City, Mexico), water ad libitum, and a constant temperature of 21 °C, with 12 h of light and 12 h of darkness. They were randomly divided into six experimental groups (*n* = 12); After O_3_ exposure, the animals were deeply anesthetized with sodium pentobarbital at a dose of 50 mg/kg, as described in NOM-033-SAG-ZOO-2014 guidelines [35]. The tissues of six subjects from each group were kept frozen at −80 °C for TBARS and Western blot techniques, while the samples from the other six animals (duodenum, jejunum, and colon) were collected and fixed in 10% formaldehyde for immunohistochemical and histologic techniques.

### 2.1. Exposure to O_3_

Animal exposure to O_3_ was carried out for 4 h daily following the methodology of Pereira et al., 2006 [36] and Rivas-Arancibia et al., 2010 [15]. Briefly, an air compressor (5 L/s) connected to an O_3_ generator was used, which supplied 0.25 parts per million (ppm) of O_3_ constantly. O_3_ levels were monitored throughout the experiment with an O_3_ monitor (PCI O_3_ and Control Systems, West Caldwell, NJ, USA). A control group was exposed to air free of O_3_, and the other groups received one of the following treatments of O_3_ for 7, 15, 30, 60, and 90 days, respectively.

### 2.2. TBARS

To evaluate oxidative stress levels, malondialdehyde (MDA) content was measured using the thiobarbituric acid reactive substances (TBARS) test. Tissues were removed and washed with PBS solution. Tissue fragments were homogenized in a mixture of PBS + Butylhydroxytoluene (Sigma 34750, St. Louis, MO, USA) (5 mM) in 1 mL of acetonitrile and centrifuged at 5000× *g* for 3 min at 4 °C. Supernatants were recovered, and total protein quantification was performed using Micro BCA (Thermo Scientific 23235, Waltham, MA, USA). Then, 100 µL of each tissue was incubated in a 1:1 solution of sulfosalicylic acid (3%) at 4 °C overnight. The samples were resuspended and centrifuged at 11,000 rpm for 3 min. An amount of 190 μL of thiobarbituric acid (TBA) solution, prepared with 4% TBA, 20% trichloroacetic acid, and HCl, was added to 10 μL of the supernatant. The mixture was incubated at 95 °C for 60 min. The reaction was measured at 532 nm in a plate spectrophotometer (Biotek, Winooski, VT, USA). Tetraethoxypropane (Sigma T-9889, St. Louis, MO, USA) was used as a standard to make the calibration curve [37].

### 2.3. Western Blot

To determine the relative content of haptoglobin and the interleukins IL-1β and IL-6, Western blot assays were performed on duodenum, jejunum, and colon tissue. Samples were processed in a protein lysis buffer supplemented with a protease inhibitor cocktail (Roche 11836170001, Basel, Switzerland) and then quantified using a protein quantification kit (Thermo Scientific 23235, Waltham, MA, USA). 100 µg of protein for IL-1β and IL-6, and 40 µg of protein for haptoglobin were separated by electrophoresis in sodium dodecyl sulfate polyacrylamide gels (SDS-PAGE 12% and 10%, respectively). The membranes were then transferred to PVDF (Millipore Sigma ISEQ00010, Burlington, MA, USA). Membranes were blocked with 5% skim milk powder in TBST (TBS + 0.01% Tween 20) for 60 min at room temperature. The following antibodies were used: against haptoglobin (sc-390962, Santa Cruz Biotechnology, Dallas, TX, USA) at 1:1000; against IL-1β (sc-7884, Santa Cruz Biotechnology, USA) at 1:1000, and against IL-6 (ab6672, Abcam, Cambridge, MA, USA) at 1:1000. The membranes were left overnight at 4 °C. As a loading control, an antibody against β-actin (GTX110564, Genetex, Irvine, CA, USA) at a dilution of 1:1000 was used under the same incubation conditions. The membranes were rinsed with TBST and subsequently incubated with horseradish peroxidase-conjugated anti-rabbit IgG (sc-2357, Santa Cruz Biotechnology USA, for IL-1β and IL-6) and anti-mouse IgG (sc-2005, Santa Cruz Biotechnology USA, for haptoglobin) for 1 h at 1:10,000, followed by three washes with TBST. PVDF membranes were stained with three antibodies (actin, IL-1β, and IL-6), washed with a low-pH glycine–HCl buffer (Gly-HCl, 0.1 M, pH 2.2) to remove antibodies from the membrane, and then reused. Different molecular weight antibodies were utilized to reuse membranes following a stripping protocol. The membranes were developed using Immobilon^®^ Forte Western HRP Substrate reagent (Millipore WBLUF0500, Burlington, MA, USA), and images were digitized using GelCapture software (v 7.0.5, DNR Bio Imaging System, Lincolnshire, IL, USA). Band density was read using Image Studio software (v 5.2.5, LI-COR Bioscience, Lincoln, NE, USA).

### 2.4. Immunohistochemistry

To study the localization of haptoglobin, IL-1β, and IL-6, immunohistochemistry experiments were performed in the duodenum, jejunum, and colon. The tissues were dehydrated and embedded in paraffin blocks, and 5-µm-thick cross-sections were made and mounted on slides. The tissues were deparaffinized and hydrated, and an antigen retrieval reagent (Biocare Medical DV2004, Pacheco, CA, USA) was used. Peroxidase activity was inhibited with 3% H_2_O_2_, and blocking was performed to reduce background (Background Sniper, 4plus Detection, Biocare Medical, BS966, USA). To identify the localization of haptoglobin, the slides were incubated overnight at 4 °C with antibodies against haptoglobin (sc-390962, Santa Cruz Biotechnology, USA) at 1:200 dilution, and against IL-6 (ab6672, ABCAM, USA) at 1:200 dilution, and IL-1β (sc-7884, Santa Cruz Biotechnology, USA) at 1:200 dilution. They were incubated with biotinylated secondary antibody (Biocare Medical STU700, USA). Streptavidin (Biocare Medical STHRP700, USA) was then used, and the slides were developed with 3,3-diaminobenzidine substrate chromogen (Biocare Medical DS854H, USA) and counterstained with hematoxylin. Each slide was analyzed with an Olympus BX41 microscope (Olympus, Tokyo, Japan), and photographs were taken with a digital camera (Evolution-QImagin MediaCybernetics, Rockville, MD, USA).

### 2.5. Hematoxylin and Eosin (H-E) Histological Technique

The sections were placed on slides, and the tissues were subsequently rehydrated in a train using xylene for 2 min, followed by re-immersion in a second bath of xylene for another 2 min. Next, tissues were passed through a train of alcohols: 100%, 96%, 70%, and 50% for 2 min at each concentration. Finally, they were washed with distilled water. The slides were then immersed in hematoxylin for 3 min, rinsed with running water for 1 min, placed in alcohol for 1 min, and washed with water for 1 min. They were then immersed in 2% eosin for 45 s. The slides were then dehydrated and rinsed in 95% alcohol for 1 min, followed by absolute alcohol for 2 min. This step was repeated, and then the slides were placed in xylene for 2 min, followed by another 2 min in xylene. Finally, mounting medium was applied to each sample, and a coverslip was placed over it to be observed under an Olympus BX41 microscope (Olympus, Japan). Photographs were then taken using a digital camera (Evolution-QImagin MediaCybernetics, USA).

### 2.6. Statistical Analysis

Western blot data were analyzed using the Kolmogorov–Smirnov normality test. The data were subsequently analyzed using the Kruskal–Wallis test to compare all groups. Mann–Whitney U tests were performed to compare the control group with each of the treated groups. Differences were considered statistically significant if *p* ≤ 0.05. All statistical analyses were performed using GraphPad Prism^®^ version 5.00 (GraphPad Software, San Diego, CA, USA). TBARS and Western blot results are presented as medians and interquartile ranges for non-parametric variables. Western blot data were analyzed using the Kolmogorov–Smirnov normality test. The data were subsequently analyzed using the Kruskal–Wallis test to compare all groups. Mann–Whitney U tests were performed to compare the control group with each of the treated groups. Differences were considered statistically significant if *p* ≤ 0.05. All statistical analyses were performed using GraphPad Prism^®^ version 5.00 (GraphPad Software, USA). TBARS and Western blot results are presented as medians and interquartile ranges for non-parametric variables. Each figure shows representative data from each experiment (*n* = 6).

## 3. Results

### 3.1. Oxidative Stress Status in the Duodenum, Jejunum, and Colon of the Rat

**Figure 1:**
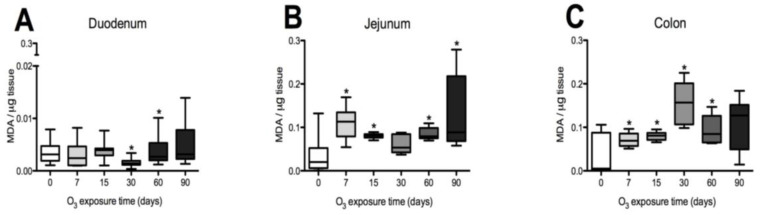
Effect of O_3_ exposure on lipid oxidation in the duodenum, jejunum, and colon of rats. The *x*-axis shows the duration of exposure to O_3_, and the *y*-axis shows MDA levels in µM MDA/µg tissue. In the duodenum, the results show a significant decrease in MDA at 30 days and an increase at 60 days compared to the control group (**A**). In the jejunum, there was a significant increase in MDA at 7, 15, 60, and 90 days of exposure to O_3_ compared to the control group (**B**). In the colon, statistically significant increases in MDA were observed at 7, 15, 30, and 60 days compared to the control group (**C**). * *p* ≤ 0.05.

To evaluate the effects of O_3_ exposure on ROS, lipid oxidation assays were performed in the three specific regions of the intestine. In the duodenum, a significant decrease in MDA levels was observed at 30 days, followed by an increase at 60 days of exposure when compared to the control group (see Figure 1A). The jejunum exhibited significant increases in the amounts of oxidized lipids at 7, 15, 60, and 90 days compared to the control group (refer to Figure 1B). In the colon, elevated levels of oxidized lipids were detected at 7, 15, 30, and 60 days in comparison to the control group (Figure 1C).

### 3.2. Western Blot Results in Duodenum, Jejunum, and Colon

#### Haptoglobin Content in Rat Duodenum, Jejunum, and Colon

**Figure 2:**
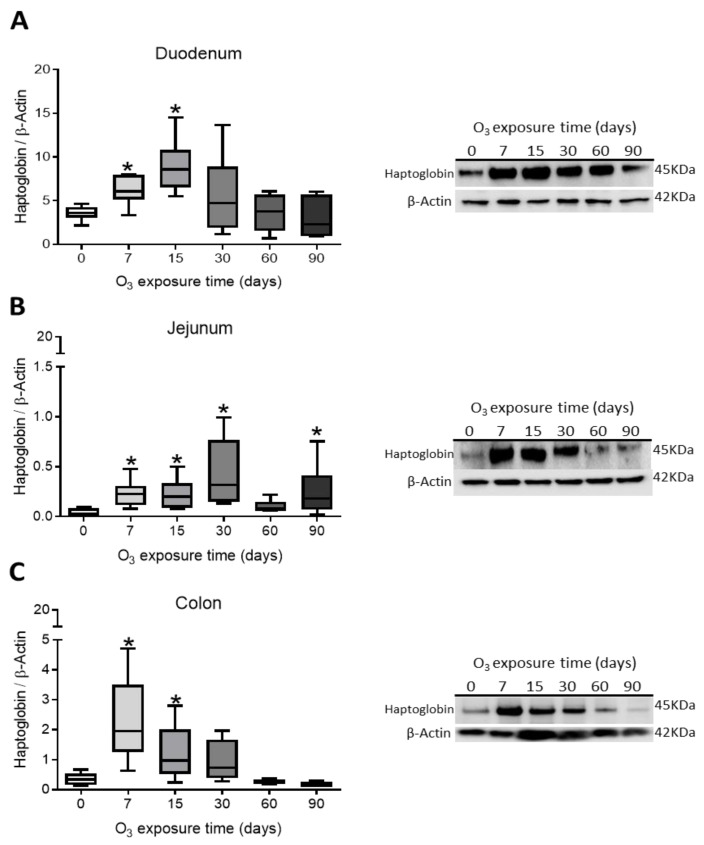
Effect of O_3_ exposure on haptoglobin content in the duodenum, jejunum, and colon of rats. The *x*-axis shows the time of exposure of animals to O_3_, and the *y*-axis shows the relative haptoglobin content expressed in arbitrary units. β-Actin was used as a loading control. The results in the duodenum show a significant increase in haptoglobin levels at 7 and 15 days compared to the control group (**A**). In the jejunum, there is a significant increase in haptoglobin at 7, 15, 30, and 90 days of exposure to O_3_ compared to the control group (**B**). In the colon, haptoglobin levels increased at 7 and 15 days compared to the control group (**C**) (* *p* < 0.05).

To evaluate the effects of different low-dose O treatments on tight junctions and to observe changes in intestinal permeability and immune response, haptoglobin content was determined. The results indicate that in the duodenum there is a significant increase in haptoglobin at 7 and 15 days of exposure to O_3_ (Figure 2A); in the jejunum, a significant increase is presented at 7, 15, 30, and 90 days of exposure to (Figure 2B); and in the colon, increases in haptoglobin are observed at 7 and 15 days of exposure to O_3_ (Figure 2C) compared to their respective control groups (*p* < 0.05).

### 3.3. L-1β Content in the Duodenum, Jejunum, and Colon of Rats

**Figure 3:**
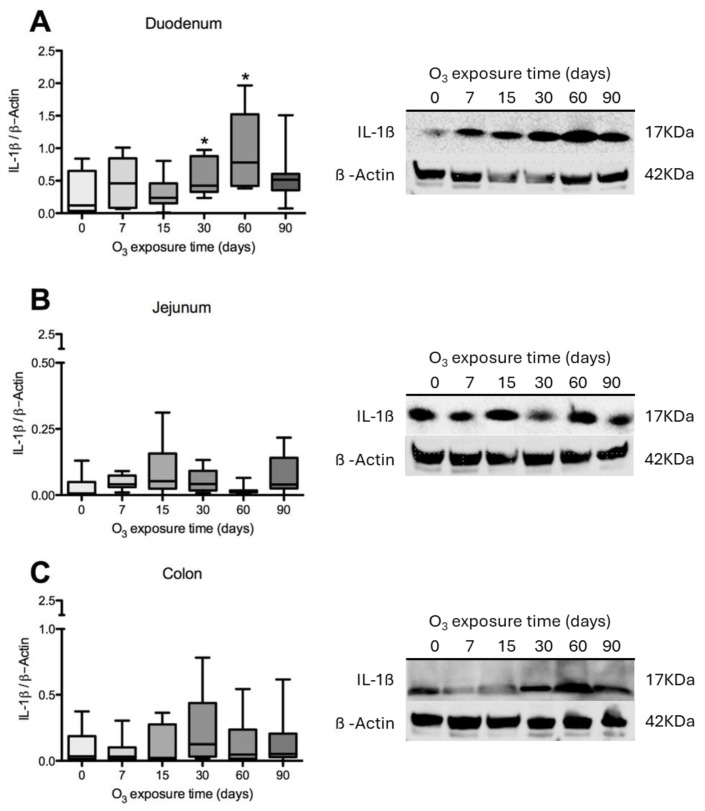
Effect of O_3_ exposure on IL-1β content in the duodenum, jejunum, and colon of rats. The *x*-axis shows the time of exposure of animals to O_3_, and the *y*-axis shows the relative content of interleukin IL-1β, expressed in arbitrary units. β-Actin was used as a loading control. The results show an increase in IL-1β in the duodenum at 30 days compared to the control group (**A**). In the jejunum, no statistically significant results were found when compared to the control group (**B**). In the colon, a statistically significant increase in IL-1β was observed at 30 days of exposure compared to the control group (**C**). * *p* ≤ 0.05.

To evaluate the effects of low-dose O_3_ treatments on the immune response, IL-1β content was determined in different regions of the intestine. The results show that the duodenum showed a significant increase after 60 days of exposure to O_3_ (Figure 3A). The jejunum did not show significant results (Figure 3B), and the colon did after 30 days of exposure to O_3_ (Figure 3C), compared to the control groups.

### 3.4. IL-6 Content in Rat Duodenum, Jejunum, and Colon

**Figure 4:**
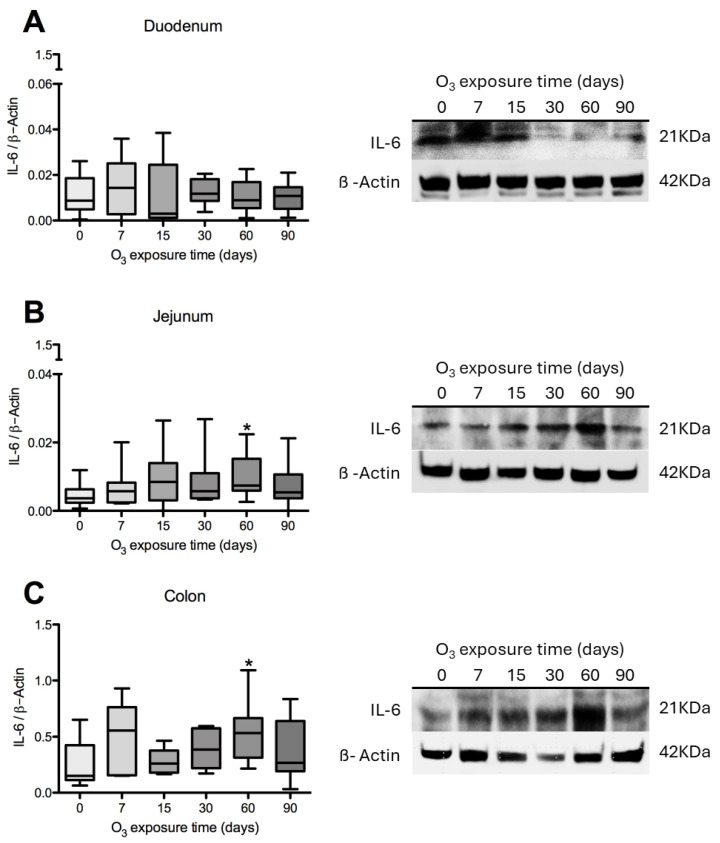
Effect of O_3_ exposure on IL-6 content in the duodenum, jejunum, and colon of rats. The *x*-axis shows the time of exposure of animals to O_3_, and the *y*-axis shows the relative IL-6 content, expressed in arbitrary units. β-Actin was used as a loading control. The results in the duodenum (**A**), jejunum (**B**), and colon (**C**) did not show significant differences when compared with their respective control groups. * *p* ≤ 0.05.

IL-6 content was assessed in the three intestinal regions. The results indicate no statistically significant differences in the duodenum (Figure 4A), jejunum (Figure 4B), and colon (Figure 4C) compared to their respective control groups.

### 3.5. Immunohistochemical Test Results

#### Immunohistochemistry Against Haptoglobin

Haptoglobin in the Duodenum

**Figure 5:**
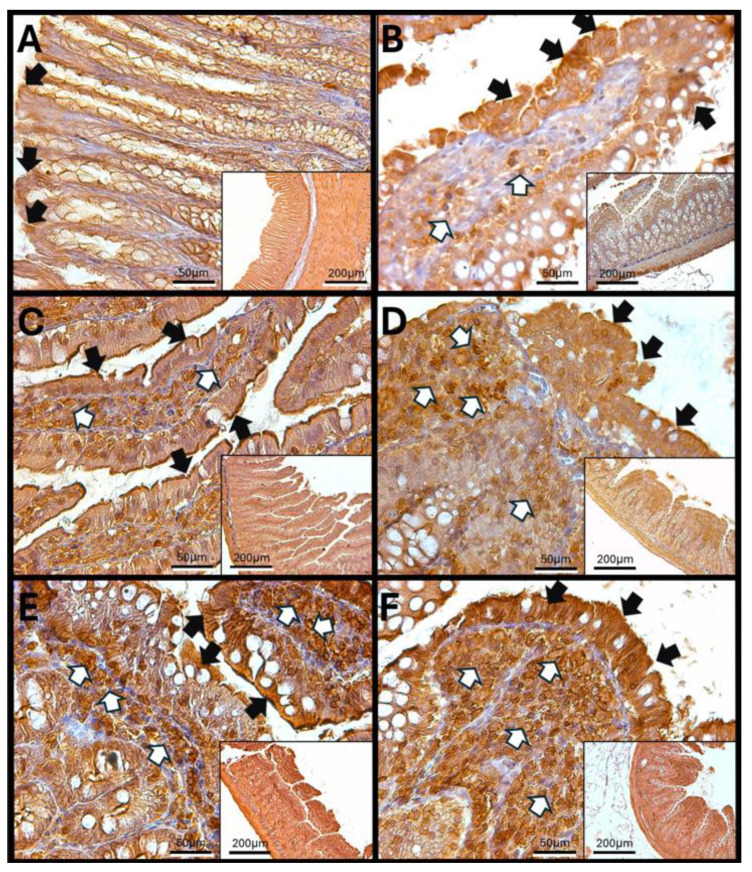
Effect of chronic exposure to low doses of ozone on haptoglobin immunoreactivity in the duodenum of rats. (**A**) Control group, (**B**) 7 days, (**C**) 15 days, (**D**) 30 days, (**E**) 60 days, and (**F**) 90 days post-treatment. The black arrows (🡆) indicate immunoreactivity in the simple columnar epithelium, while the white arrows (⇨) mark immunoreactivity in the cells of the lamina propria of the duodenum. Notably, there is an increase in immunoreactivity in both regions at 30 days (**D**), 60 days (**E**), and 90 days (**F**), whereas a decrease is observed at 15 days (**C**) compared to the control group. The micrographs are presented at magnifications of 40× (scale bar = 50 µm) and 10× (scale bar = 200 µm), respectively.

The analysis of duodenal images after exposure to O_3_ reveals that the villi in control animals show immunoreactivity against haptoglobin primarily at the edges and in the cells of the lamina propria. These villi maintain a regular shape and exhibit a parallel arrangement of Brunner’s glands. However, after 7 days of exposure, there is an increase in immunoreactive labeling within the lamina propria. By 15, 60, and 90 days, aggregates of immune system cells become apparent, and their immunoreactivity against haptoglobin increases. In contrast to the control animals, the enterocytes in the exposed animals display intense labeling in the cytoplasm. Additionally, the continuity of the epithelium is disrupted, and the arrangement of goblet cells becomes disorganized (Figure 5).

### 3.6. Haptoglobin in the Jejunum

**Figure 6:**
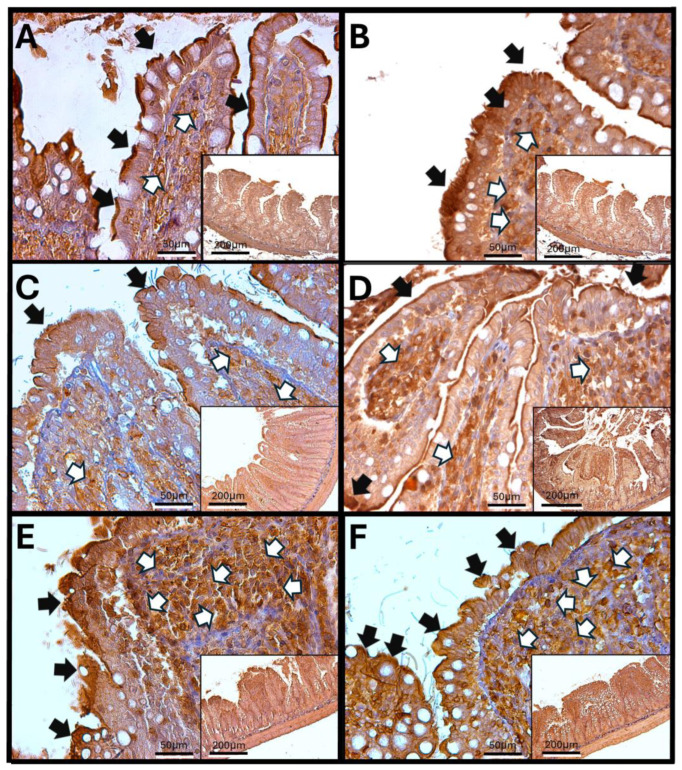
Effect of chronic exposure to low doses of ozone on haptoglobin immunoreactivity in the jejunum of rats. (**A**) Control group, (**B**) 7 days, (**C**) 15 days, (**D**) 30 days, (**E**) 60 days, and (**F**) 90 days after treatment. The black arrows (🡆) indicate immunoreactivity in the simple columnar epithelium, and the white arrows (⇨) indicate immunoreactivity in the cells of the lamina propria of the jejunum. Note the increase in immunoreactivity in both regions in (**C**–**F**), as well as a decrease at 7 days (**C**) compared to the control. The micrographs are shown at magnifications of 40× (bar = 50 µm) and 10× (bar = 200 µm), respectively.

Haptoglobin is found on the surface of enterocytes and in the lamina propria cells of the jejunum, similar to its presence in the duodenum. In control animals, immunoreactivity can be observed in the cells surrounding the crypts of Lieberkühn. However, in specimens exposed to O_3_, there is an increase in the number of haptoglobin-immunoreactive cells within the lamina propria. The infiltration of immune system cells and changes in the intestinal epithelium’s structure mark this condition. Additionally, after 30, 60, and 90 days of O_3_ exposure, alterations in the arrangement of enterocytes are evident, along with a more intense intracellular immunoreactive signal. This pattern differs from that seen in epithelial cells exposed to O_3_ for shorter periods, where haptoglobin is primarily detected in the apical region of the enterocytes (Figure 6).

### 3.7. Haptoglobin in the Colon

**Figure 7:**
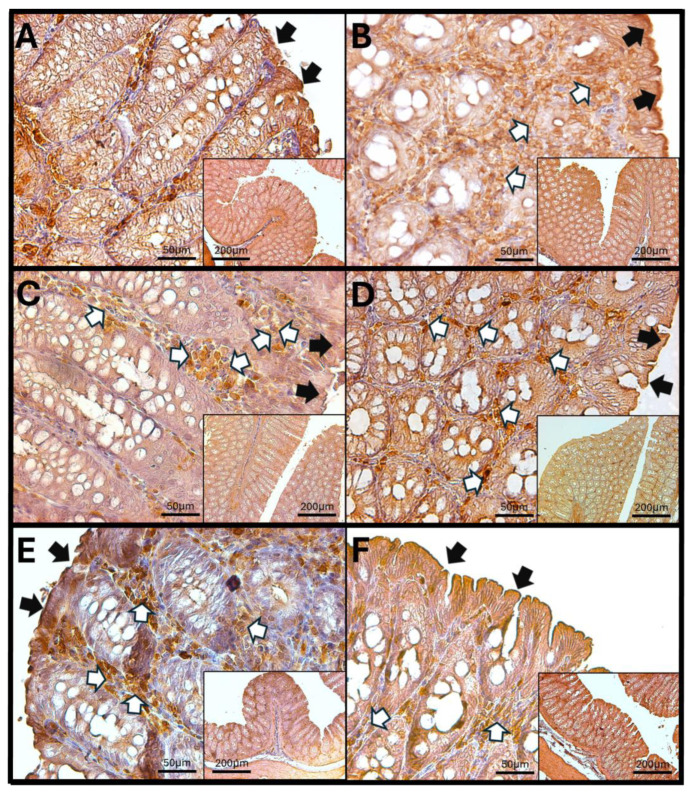
Effect of chronic exposure to low doses of ozone on haptoglobin immunoreactivity in the colon of rats. (**A**) Control group, (**B**) 7 days, (**C**) 15 days, (**D**) 30 days, (**E**) 60 days, and (**F**) 90 days after treatment. The black arrows (🡆) indicate immunoreactivity in the simple columnar epithelium, and the white arrows (⇨) indicate immunoreactivity in the cells of the lamina propria of the colon. Note the increase in immunoreactivity in both regions in E, as well as an increase in immunoreactivity in the lamina propria compared to the control. Micrographs (**B**–**D**) show no changes. The micrographs are shown at magnifications of 40× (bar = 50 µm) and 10× (bar = 200 µm), respectively.

Haptoglobin is located in the apical zone of colonic enterocytes in the colon, where it regulates the function of intestinal tight junctions. An increase in the number of cells in the lamina propria that exhibit immunoreactivity against haptoglobin was observed after 60 and 90 days of O_3_ exposure. Additionally, after 90 days of exposure, a noticeable discontinuity in the colonic epithelium was found, indicating that repeated exposure to O_3_ significantly impacts epithelial tight junctions and may have adverse effects on the overall function of the gastrointestinal system (Figure 7).

### 3.8. Immunohistochemistry Against IL-1β

#### 3.8.1. IL-1β in Rat Duodenum

**Figure 8:**
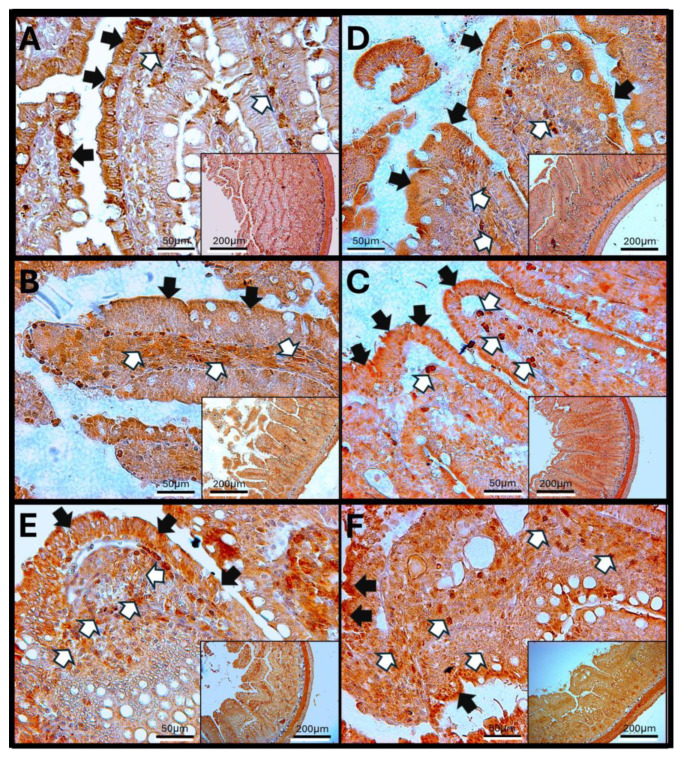
Effect of chronic exposure to low doses of ozone on IL6 immunoreactivity in the duodenum of rats. (**A**) Control group, (**B**) 7 days, (**C**) 15 days, (**D**) 30 days, (**E**) 60 days, and (**F**) 90 days after treatment. The black arrows (🡆) indicate immunoreactivity in the simple columnar epithelium, and the white arrows (⇨) indicate immunoreactivity in the cells of the lamina propria of the duodenum. Note the increase in immunoreactivity in both regions in (**B**–**D**,**F**), as well as a decrease in immunoreactivity in the lamina propria in (**E**). Micrographs are shown at a magnification of 40× (bar = 50 µm) and 10× (bar = 200 µm), respectively.

The duodenum of control animals (A) shows IL-1β staining in enterocytes, as well as around the crypts and Brunner’s glands. With increasing exposure time to O_3_, a significant rise in the staining signal is observed in the lamina propria of the tissue, which increases from 7 to 90 days of exposure to O_3_. Additionally, changes in the structure of the duodenal villi and infiltration of immune cells are noted. IL-1β staining is also present in the muscular layer of the duodenum across the different experimental groups. These findings suggest a more comprehensive and complex immune response (Figure 8).

#### 3.8.2. IL-1β in the Rat Jejunum

**Figure 9:**
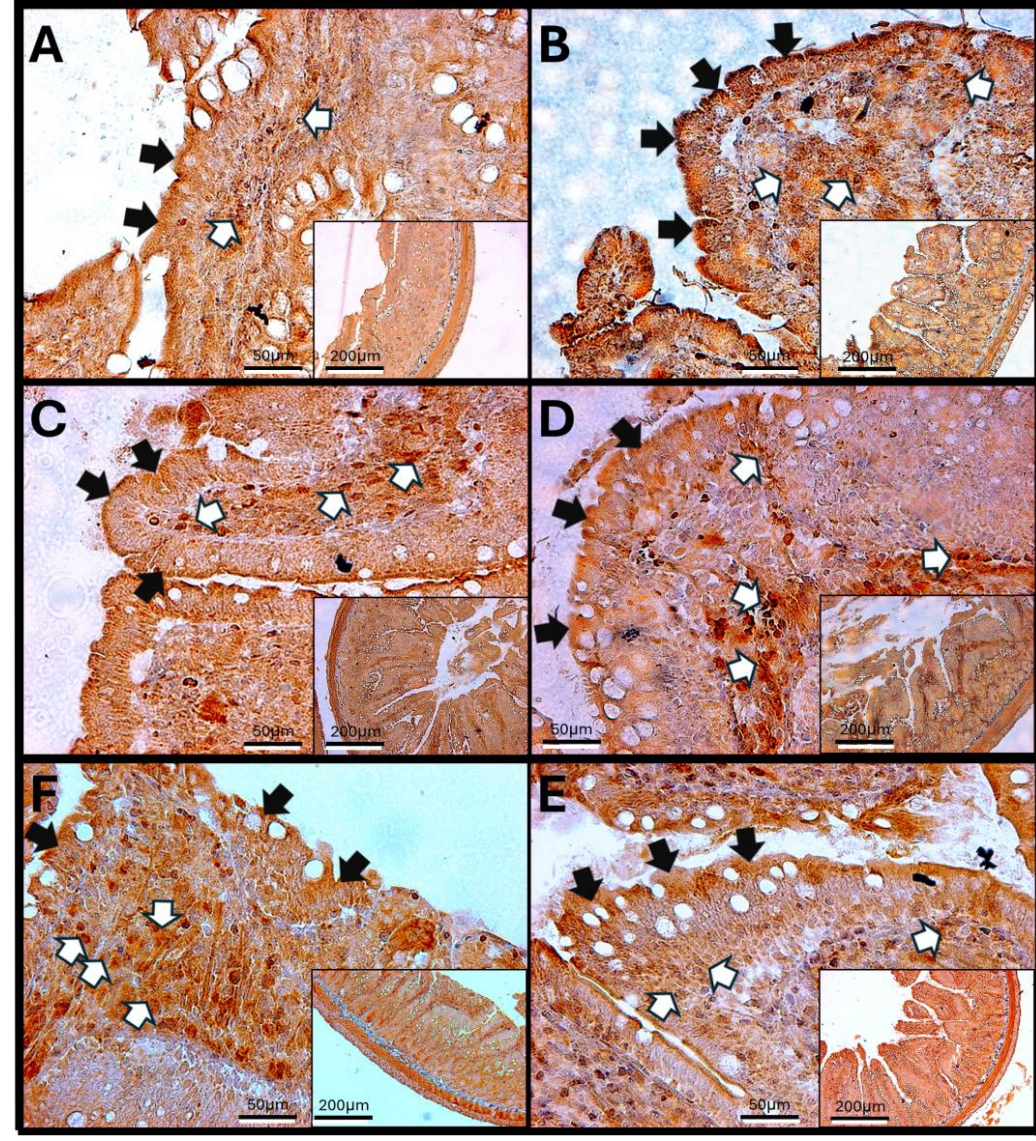
Effect of chronic exposure to low doses of ozone on IL6 immunoreactivity in the jejunum of rats. (**A**) Control group, (**B**) 7 days, (**C**) 15 days, (**D**) 30 days, (**E**) 60 days, and (**F**) 90 days after treatment. The black arrows (🡆) indicate immunoreactivity in the simple columnar epithelium, and the white arrows (⇨) indicate immunoreactivity in the lamina propria cells of the jejunum. Note the decrease in immunoreactivity in the columnar epithelium in (**B**,**F**), as well as an increase in immunoreactivity in both regions in (**C**,**D**). In (**E**), a decrease in immunoreactivity is observed in the lamina propria cells with respect to the control. The micrographs are shown at magnifications of 40× (bar = 50 µm) and 10× (bar = 200 µm), respectively.

IL-1β in the rat jejunum shows immunoreactive signals in the enterocytes and cells of the lamina propria. In addition to the structural changes observed in the intestinal villi of the jejunum, there are increases in IL-1β levels in the lamina propria during the exposure periods, particularly on days 15, 30, and 90 of exposure to O_3_. Furthermore, the muscular layer exhibits strong reactivity to IL-1β at various exposure times, indicating an inflammatory response (Figure 9).

#### 3.8.3. IL-1β in the Rat Colon

**Figure 10:**
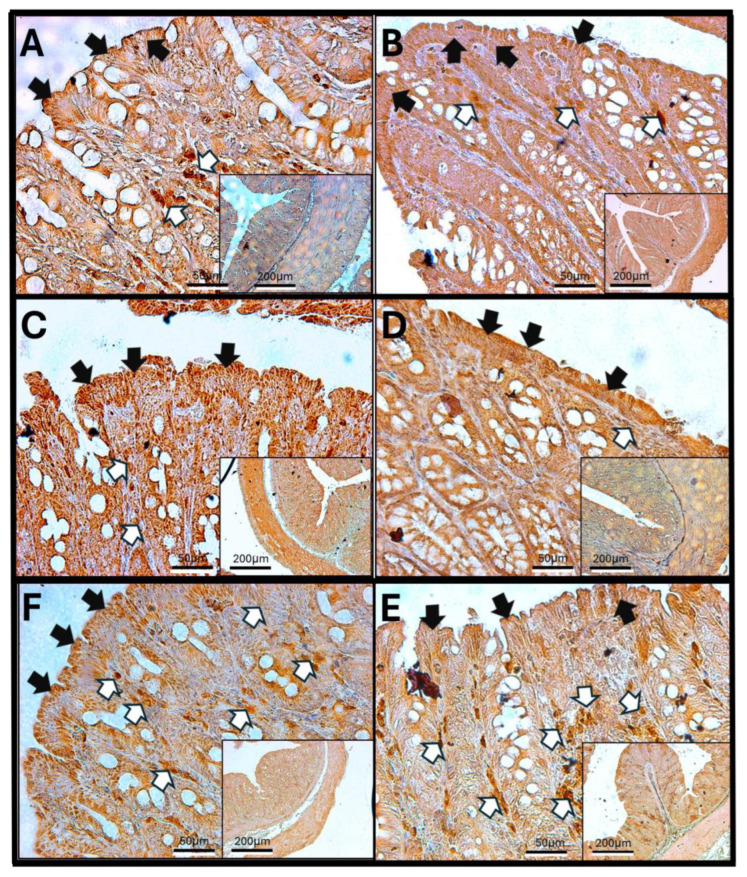
Effect of chronic exposure to low doses of ozone on IL6 immunoreactivity in the colon of rats. (**A**) Control group, (**B**) 7 days, (**C**) 15 days, (**D**) 30 days, (**E**) 60 days, and (**F**) 90 days after treatment. The black arrows (🡆) indicate immunoreactivity in the simple columnar epithelium, and the arrows (⇨) indicate immunoreactivity in the lamina propria cells of the colon. Note a decrease in immunoreactivity in both the simple columnar epithelium and the lamina propria cells in (**B**), as well as a decrease in the columnar epithelium in (**E**) with respect to the control. Micrographs are shown at magnifications of 40× (bar = 50 μm) and 10× (bar = 200 μm), respectively.

The results of IL-1β in the rat colon are presented in Figure 10. The micrographs illustrate the presence of IL-1β-reactive cells after exposure to O_3_ in the rat colon. IL-1β is distributed in cells within the lamina propria, as well as in enterocytes. An increase in immunoreactivity is observed around the crypts at 15 and 30 days. By 60 and 90 days, disruptions in the composition of the colonic epithelium become evident.

### 3.9. Immunohistochemistry Against IL-6

**Figure 11:**
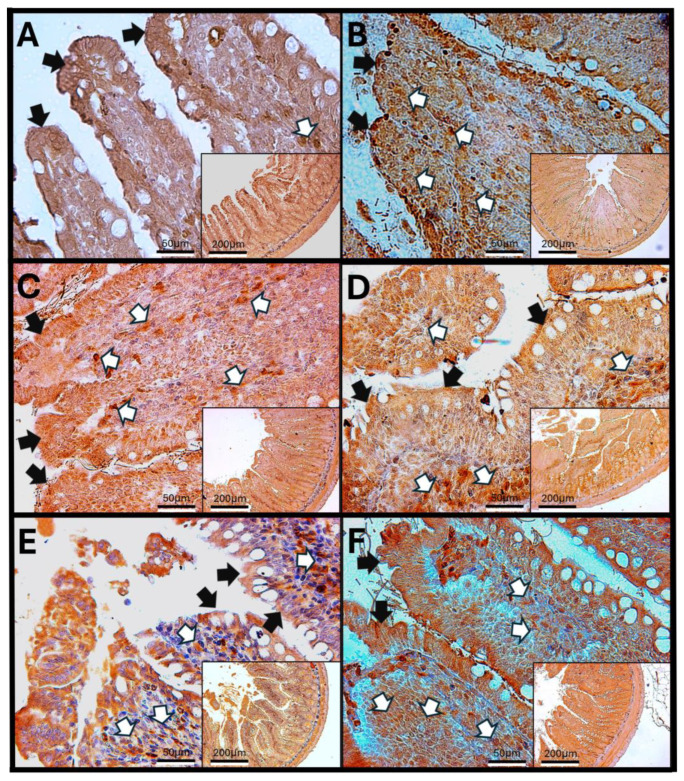
Effect of chronic exposure to low doses of ozone on IL-1β immunoreactivity in the duodenum of rats. (**A**) Control group, (**B**) 7 days, (**C**) 15 days, (**D**) 30 days, (**E**) 60 days, and (**F**) 90 days after treatment. The black arrows (🡆) indicate immunoreactivity in the simple white columnar epithelium, and the arrows (⇨) indicate immunoreactivity in the lamina propria cells of the duodenum. Note a decrease in immunoreactivity in the columnar epithelium in (**B**), as well as an increase in immunoreactivity in the lamina propria cells in (**B**–**F**) with respect to the control. The micrographs are shown at magnifications of 40× (bar = 50 μm) and 10× (bar = 200 μm), respectively.

Immunohistochemistry for IL-6 was conducted on the duodenum of rats. The results indicate that IL-6 immunoreactivity is present in enterocytes and lamina propria cells of control rats. In animals exposed to O_3_ for 7, 15, 30, 60, and 90 days, noticeable alterations in the villi structure and epithelial surface were observed. Additionally, disruptions in the arrangement of goblet cells were evident after 15, 30, and 90 days of O_3_ exposure (Figure 11).

### 3.10. IL-6 in Rat Jejunum

**Figure 12:**
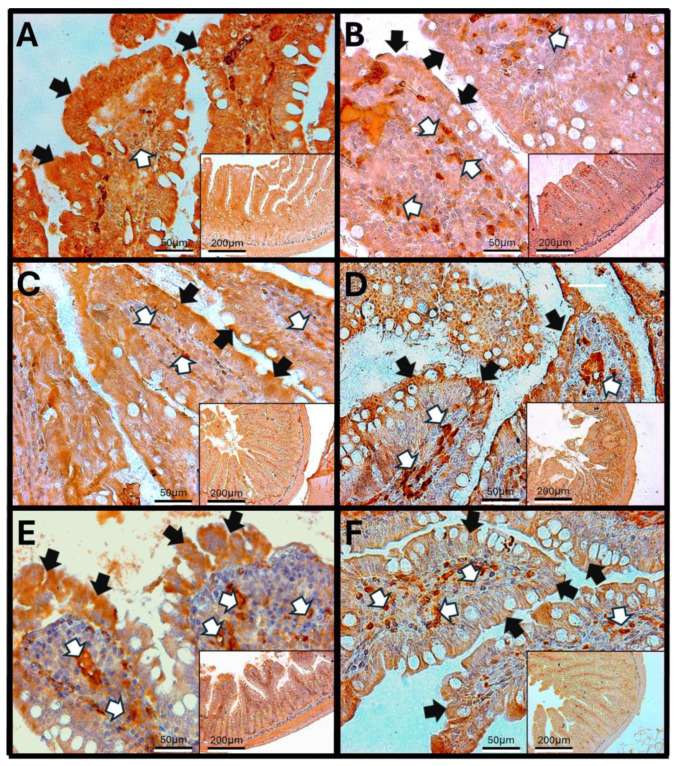
Effect of chronic exposure to low doses of ozone on IL-1β immunoreactivity in the jejunum of rats. (**A**) Control group, (**B**) 7 days, (**C**) 15 days, (**D**) 30 days, (**E**) 60 days, and (**F**) 90 days after treatment. The black arrows (🡆) indicate immunoreactivity in the simple columnar epithelium, and the arrows (⇨) indicate immunoreactivity in the lamina propria cells of the jejunum. Note an increase in immunoreactivity in the simple columnar epithelium in (**B**), as well as an increase in immunoreactivity in the lamina propria cells in (**B**–**E**), with respect to the control. The micrographs are shown at magnifications of 40× (bar = 50 μm) and 10× (bar = 200 μm), respectively.

In the jejunum of rats, the results show that control animals exhibit IL-6 immunoreactivity in both enterocytes and lamina propria cells. After 7, 30, and 60 days of exposure to O_3_, there was noticeable cell infiltration and an increase in IL-6-reactive cells within the lamina propria. Additionally, alterations in the structure of the intestinal villi and significant changes in the epithelial layer were observed (Figure 12).

### 3.11. IL-6 Immunoreactivity in the Rat Colon

**Figure 13:**
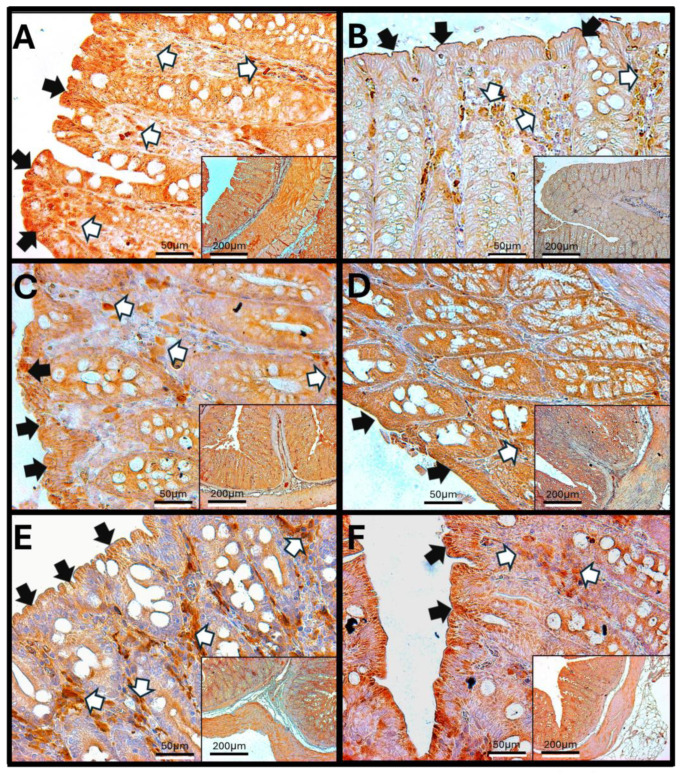
Effect of chronic exposure to low doses of ozone on IL-1β immunoreactivity in the colon of rats. (**A**) Control group, (**B**) 7 days, (**C**) 15 days, (**D**) 30 days, (**E**) 60 days, and (**F**) 90 days after treatment. The black arrows (🡆) indicate immunoreactivity in the simple columnar epithelium, and the white arrows (⇨) indicate immunoreactivity in the lamina propria cells of the colon. Note an increase in immunoreactivity in the simple columnar epithelium in (**B**–**D**), as well as a decrease in immunoreactivity in the lamina propria cells in (**B**,**D**) with respect to the control. The micrographs are shown at magnifications of 40× (bar = 50 µm) and 10× (bar = 200 µm), respectively.

The results of IL-6 immunoreactivity in the rat colon show variations depending on the duration of exposure to O_3_. IL-6 is detected in enterocytes and the lamina propria, with its expression increasing at 60 and 90 days of treatment compared to control animals. Additionally, there is a noted loss of epithelial continuity (Figure 13).

### 3.12. Results of H-E Staining in the Duodenum, Jejunum, and Colon of the Rat

**Figure 14:**
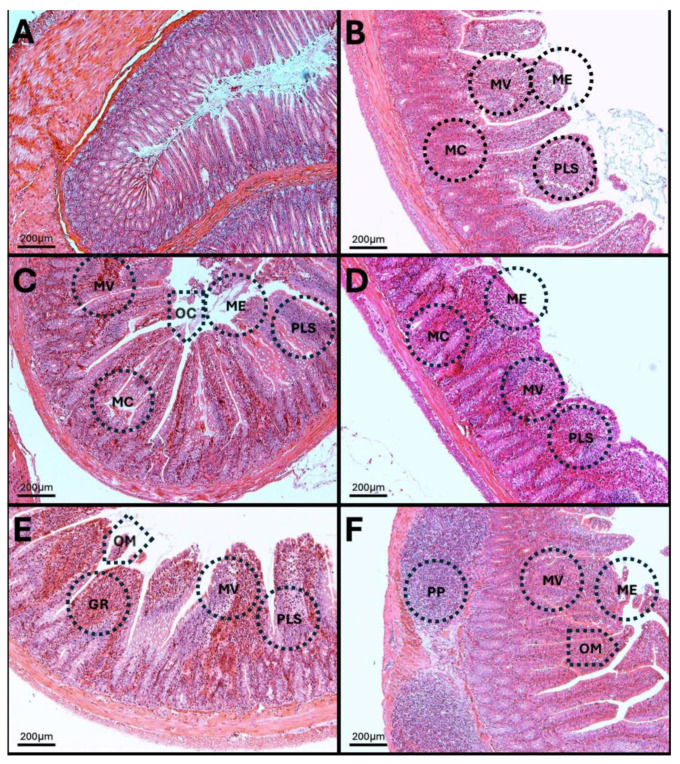
Cellular and structural changes in the duodenum of rats exposed to low doses of O_3_. The micrographs show cellular and structural changes in the duodenum of rats chronically exposed to O_3_. (**A**) Control group without O_3_. Tall villi with unaltered columnar epithelium, in the form of a continuous brush; there are no erosions or loss of continuity. The stroma of the lamina propria appears normal, with few, sparse, and scattered cells. Mucin cells are abundant and uniform. (**B**) O_3_ group at 7 days. The subepithelial region shows plasmacytosis (PLS) and vacuoles, which modify the structure of the villi (MV); the epithelial contour is modified (ME). Mucin cells (MC) appear sparse compared to the control group, and some crypts are elongated. (**C**) O_3_ group at 15 days. Foci of epithelial erosion and opening of the crypt mouths are visible (OM). Infiltrated lamina propria. Mucin cells are seen in smaller numbers and located toward the surface of the epithelium. (**D**) O_3_ group, 30 days old. The epithelium appears cuboidal due to fused villi with a discontinuous surface. Massive infiltration in the lamina propria is observed. Mucin cells are scarce. (**E**) O_3_ group, 60 days old. Opening of the crypt mouths is observed. The lamina propria shows marked plasmacytosis (PLS) and granulomas (GR). Isolated, deformed, and small mucous cells are present. (**F**) O_3_ group, 90 days old. Columnar epithelium is partially restored, and the crypt mouths are seen wide; there are signs of epithelial erosion. Residual infiltration in the villi and Peyer’s patches (PP) are observed in the region. Mucin cells are reorganized into a glandular shape. Each photograph shows the area at 10× (bar = 200 µm).

H-E of the duodenum. In the histology of the duodenum of control animals, the structure of the villi is normal with a parallel arrangement, and no alterations are observed in the lamina propria or Brunner’s glands. However, at 7, 15, 30, 60, and 90 days of exposure to O_3_, atrophy and loss of villous arrangement are observed as a result of infiltration of immune system cells and distortion in the architecture of goblet cells, in addition to significant alterations in the intestinal epithelium and widening at the mouth of the crypts (Figure 14).

### 3.13. H-E in Jejunum

**Figure 15:**
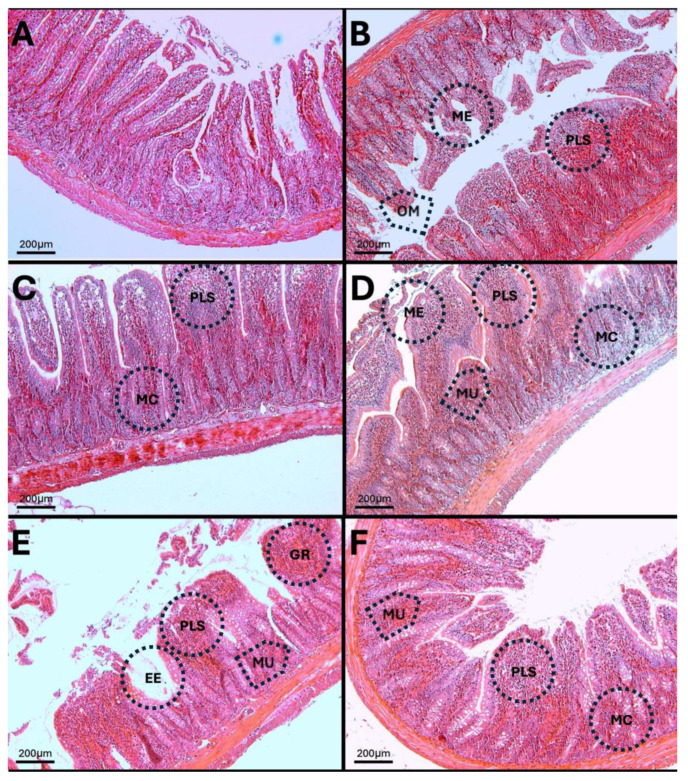
Cellular and structural alterations in the jejunum of rats exposed to low doses of O_3_. The micrographs show cellular and structural alterations in the jejunum of rats chronically exposed to O_3_. (**A**) Control group without O_3_. Tall villi, continuous contour; intact brush border; no evident erosions. Lamina propria stroma with few cells. Abundant and regular mucin cells. (**B**) Seven-day O_3_ group. Epithelial contour modified by villous fusion (ME). Subepithelial region with plasmacytosis processes (PLS). Mucin cells (MC) of unequal size. Opening of the crypt mouths are visible (OM). (**C**) Fifteen-day O_3_ group. Sites of cellular infiltration are observed in the villi. Decreased number of mucin cells, located mostly in the apical region. (**D**) Thirty-day O_3_ group. Fussed epithelium and microulcers (MU). Lamina propria with cellular infiltration. Marked decrease in mucin cells, with crypts showing altered morphology. (**E**) O_3_ group, 60 days. Highly eroded epithelium (EE). Ulcerations reaching the muscularis (MU). Massive plasmacytosis (PLS) and granulomas (GR). Small, deformed mucin glands. (**F**) O_3_ group, 90 days. Partial restoration of columnar epithelium; presence of small ulcerations (MU). Residual cellular infiltration of the lamina propria. Reappearance of mucin glands in discontinuous rows. Each photograph shows the area at 10× (bar = 200 µm).

The results show that at 7, 15, 30, 60, and 90 days of exposure to O_3_, villous atrophy and crypt distortion are evident, as well as basal plasmacytosis processes in the lamina propria (Figure 15). Additionally, erosion lesions are visible on the epithelial surface, accompanied by significant epithelial damage.

### 3.14. H-E of the Colon

**Figure 16:**
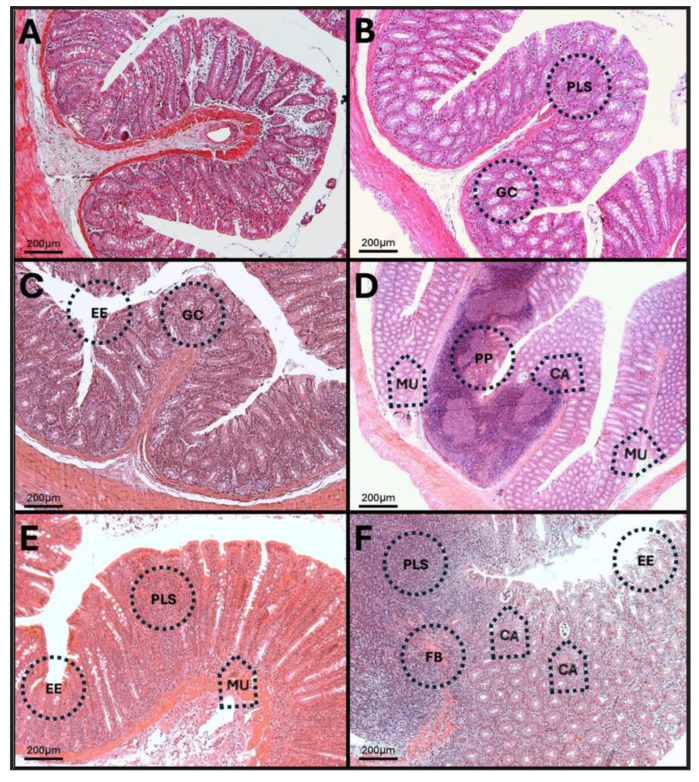
Structural changes in the colon of rats exposed to low doses of O_3_. The micrographs show structural changes in the colon of rats chronically exposed to O_3_. (**A**) Control group without O_3_. Continuous epithelial contour; no erosions. Loose stroma of the lamina propria, abundant cell count. Crypts with numerous, uniform, and dense mucin cells. (**B**) Seven-day O_3_ group. The apical contour of the epithelium is preserved. Subepithelial region with a significant number of cells in the lamina propria (PLS). Abundant mucous glands with enlarged mucin cells (MC). (**C**) Fifteen-day O_3_ group. Localized foci of epithelial erosion (EE). Increased cell infiltration into the stroma. Mucin cells hyperplasia. (**D**) Thirty-day O_3_ group. Presence of microulcers (MU). Enlargement of a Peyer’s patch with drainage into the luminal space, altering the structure and arrangement of the mucin glands; a crypt abscess is seen (CA). (**E**) O_3_ group, 60 days old. Multiple microulcers (MU) with loss of epithelial continuity; elongated crypts with numerous mucin cells. The lamina propria is undergoing plasmacytosis (PLS). (**F**) O_3_ group, 90 days old. Epithelium is undergoing erosion (EE), disrupting its continuity; a large number of infiltrating cells are reaching the luminal space; fibrosis process is seen (FB). The mucin glands are disorganized and discontinuous. Two crypt abscesses (CA) are seen.

The results show enlargement of Peyer’s patches at 30 and 90 days after low-dose O_3_ treatment (Figure 16). Also, at the same time, the formation of crypt abscesses, where eosinophils and neutrophils agglomerate, is observed. At 60 days, the loss of normal tissue structure is noticeable, as well as infiltration of immune system cells into the lamina propria.

## 4. Discussion

This study uniquely illustrates how ozone pollution impacts the intestinal barrier, potentially explaining the rise in inflammatory bowel and chronic degenerative diseases. Our primary interest was to assess the effects of ozone on the changes in the simple columnar epithelium and lamina propria cells across different intestinal segments due to oxidative stress. The intestine is considered the primary target organ of the microbiota, given its significant implications for health and the development of degenerative diseases.

This oxidation, resulting from chronic O_3_ exposure, leads to the formation of secondary products, such as ROSs, which further amplify oxidative stress and trigger inflammation. Additionally, oxidized lipids (1A, B, C) can be recognized by the immune system as pathogens through TLRs, initiating signaling cascades that activate and enhance inflammatory responses. This response contributes to the establishment of a systemic inflammatory process, as discussed by Miller & Shyy (2017) [38] and Rhoads & Major (2018) [39]. Under both normal and disease conditions, various stimuli can alter the structure of tight junctions (TJs) in the intestine. Factors that influence paracellular permeability include pathogens, growth factors, cytokines, microbiota components, substances in ingested food, digestive enzymes, oxidative stress, and proinflammatory molecules. These factors work together to regulate the opening of intestinal tight junctions [40]. When changes occur in the intestinal epithelium and innate immune cells are activated, the composition of intestinal bacterial populations may also change. These alterations can be linked to variations in mucus production, which helps eliminate pathogens and bacterial metabolites, such as short-chain fatty acids (SCFAs). SCFAs play a crucial role in various processes, including cell proliferation and differentiation, hormone secretion (such as leptin and peptide YY), and the activation of immune and inflammatory responses [41]. Therefore, maintaining a suitable microenvironment and a healthy microbiota composition are essential for the proper functioning of the intestine [42,43].

The increase in reactive oxygen species (ROSs) caused by O_3_ exposure, as demonstrated in this study (Figure 1), contributes to the recruitment and accumulation of immune system cells in the lamina propria. This process involves the synthesis and release of zonulin (Figure 5, Figure 6 and Figure 7), IL-1β (Figure 8, Figure 9 and Figure 10), and IL-6 (Figure 11, Figure 12 and Figure 13) by specific immune cells, as demonstrated by Dillon et al., 2010 [44], Shaw et al., 2012 [45], and Yonker et al., 2021 [46,47]. During the early immune response, IL-6 is primarily produced by cells of the innate immune system through the activation of TLRs. IL-6 generally enhances the production of various cytokines and chemokines, including chemokine (C-C motif) ligand 4 (CCL4), ligand 5 (CCL5), ligand 11 (CCL11), ligand 17 (CCL17), as well as intracellular and vascular adhesion molecules like intercellular adhesion molecule 1 (ICAM-1) and vascular cell adhesion molecule 1 (VCAM-1). As a result, IL-6 regulates the growth, differentiation, and circulation of CD4+ and CD8+ T cells, natural killer (NK) cells, dendritic cells, monocytes, and macrophages. This cytokine plays a vital role in signaling for leukocyte translocation and infiltration into sites of inflammation. Additionally, IL-6 promotes the differentiation of monocytes into macrophages by inducing the expression of macrophage colony-stimulating factor receptors on monocytes [48,49]. IL-1β is also crucial in the inflammatory response associated with inflammatory bowel disease (IBD) and other inflammatory conditions [50].

Zonulin, also known as prehaptoglobin-2, is a protein that plays a crucial role in the reversible disassembly of tight junctions in the intestinal epithelium, thereby regulating mucosal permeability [51]. In contrast, prehaptoglobins 1 and 2 are immature molecules; both are glycoproteins that contain a β polypeptide chain. Prehaptoglobin molecules undergo cleavage, resulting in the formation of mature haptoglobin, a glycoprotein that retains the β polypeptide chain. In this study, we utilized an antibody that recognizes the β polypeptide chain found in both types of haptoglobin, 1 and 2. Haptoglobin typically binds to hemoglobin in circulating blood. However, by using an antibody that identifies a common polypeptide chain across these molecules and showing that its immunoreactivity highlights tight tissue junctions similar to those observed in other species, we can conclude that we are detecting the mature haptoglobin present in rats. Haptoglobin, on the other hand, is part of a family of acute-phase proteins that form a complex with hemoglobin to prevent its oxidation and protect surrounding tissues [52]. It also assists in the elimination of old red blood cells in the liver. Most haptoglobin is produced in the liver, but it can also be found in the lungs, oral epithelium, and colorectal epithelium [53]. Haptoglobin is notable for having distinct biological activities in its precursor form, which differ from the functions of its mature form. In its precursor state, it is involved in the development of various human immunological diseases [54]. As a circulating protein, haptoglobin has antioxidant, anti-inflammatory, and senescent cell elimination effects.

In our research, exposure to low doses of O_3_ impacts the presence of haptoglobin in the duodenum (Figure 2A), jejunum (Figure 2B), and colon (Figure 2C), as well as its localization in the duodenum (Figure 5), jejunum (Figure 6), and colon (Figure 7). These changes are influenced by the duration of exposure and the specific intestinal structure being studied. Since zonulin is a precursor molecule of haptoglobin and is often referenced interchangeably in various studies, we used zonulin as a marker for intestinal permeability in the tissue. Zonulin modulates intestinal permeability by regulating the opening of TJs. This mechanism is crucial for the recruitment and activation of innate immune cells in the intestine. Elevated levels of zonulin are implicated in the pathogenesis of autoimmune diseases, such as celiac disease [51], and contribute to the persistence of inflammatory states. Our results indicate significant changes in haptoglobin concentration in the duodenum (Figure 2A) after 7 and 15 days of treatment. In the jejunum (Figure 2B), notable changes are observed at 7, 15, 30, and 90 days of O_3_ exposure. The colon (Figure 2C) shows a significant increase at both 7 and 15 days. Furthermore, O_3_ treatment leads to alterations in haptoglobin localization, as seen in the duodenum (Figure 5), jejunum (Figure 6), and colon (Figure 7).

A strong relationship exists between ROS and the immune system, as changes in the redox balance can lead to alterations in immune responses. Repeated exposure to low doses of O_3_ disrupts this redox balance, leading to a chronic state of oxidative stress that affects interleukins and other pro-inflammatory molecules. Previous studies conducted in our laboratory have shown that reactive oxygen species (ROS) cause the phosphorylation of NF-κB. This process leads to the increased translocation of NF-κB to the nucleus, resulting in the production of pro-inflammatory cytokines. Additionally, some research has demonstrated that changes in the translocation of Nrf2 to the nucleus occur, leading to chronic alterations that disrupt the regulation of antioxidant systems. Together, these two transcription factors highlight the complex relationship between the balance of oxidation and reduction and the inflammatory response [55,56].

The findings of this study show that both IL-1β and IL-6 experience changes in the intestine, with variations based on the region and the treatment with O_3_. Specifically, there is a significant increase in IL-1β levels in the duodenum after 60 days of treatment, while in the colon, this increase is observed after 30 days.

Examining the distribution of IL-1β in the duodenum reveals the highest immunoreactivity at 15 and 60 days across different structures, including enterocytes, the lamina propria, villi, and the muscular layer. In the jejunum, more pronounced immunoreactivity is noted at 15, 30, and 90 days compared to the control group. Finally, in the colon, there is an increase in immunoreactivity after 15 days of treatment. IL-1β is a common and versatile proinflammatory cytokine that plays a critical role in the development of intestinal inflammation. Patients with IBD typically have elevated levels of IL-1β, and it is also significant in animal models of intestinal inflammation [50,57]. Although the role of alterations in epithelial TJs in the development of intestinal inflammation associated with IBD is recognized, there are currently no therapeutic agents available that specifically target the treatment of TJs.

ROS play a crucial role in recruiting and accumulating immune cells in the lamina propria. These immune cells synthesize and release key substances, including zonulin, IL-1β, and IL-6. During the early stages of the immune response, innate immune cells activated through TLRs are the primary source of IL-6. Our experimental results indicate that IL-6 levels in the duodenum tend to increase at both 7 and 15 days post-stimulation. In contrast, significant increases in IL-6 levels are observed in the jejunum and colon at 60 days. Specifically, there is an increase in immunoreactivity at 60 and 90 days in the duodenum (see Figure 11), at 30 and 60 days in the jejunum (see Figure 12), and at 30 days in the colon (see Figure 13).

These alterations together generate alterations in the structure and function of intestinal tissue, such as modifications in tight junctions that increase intestinal permeability, distortion in the Liberkühn crypts, atrophy in intestinal villi, widening at the mouth of the crypts, erosion of the epithelium, the formation of eosinophil and neutrophil abscesses in the crypts, and often enlargement of Peyer’s patches (Figure 14, Figure 15 and Figure 16). These changes can result in altered immune responses, characterized by proinflammatory cytokines (Figure 8, Figure 9, Figure 10, Figure 11, Figure 12 and Figure 13) causing modifications in the structure of the intestinal tract with the consequent synthesis and release of zonulin (Figure 5, Figure 6 and Figure 7), to the detriment of intestinal permeability; this allows the passage of microbial debris and antigens from the luminal space to the basolateral layer, perpetuating a state of unregulated intestinal permeability, inflammation and dysbiosis, similar to what occurs in IBD, which are chronic inflammatory disorders of the gastrointestinal tract that have complex interactions between genetic predisposition, environmental factors, intestinal microbiota, and the immune system [57].

Finally, human inhalation of pollutants involves a complex mixture of suspended particles and gases, which can produce ozone in the troposphere when exposed to ultraviolet light. While some researchers have explored the effects of environmental pollution on the health of individuals exposed to these gases, they have not been able to pinpoint the specific impact of any single pollutant. As a result, there are no human studies that provide a clear understanding of the effects of ozone pollution on its own. However, using animal models allows us to investigate its effects and determine how chronic oxidative stress contributes to human health issues associated with this pollutant.

## 5. Conclusions

In conclusion, repeated exposure to low doses of O_3_ causes increased oxidative stress, an increase in proinflammatory cytokines that leads to a loss of regulation of the immune response in the duodenum, jejunum, and colon of rats, as well as structural changes that alter the intestinal barrier and perpetuate a state of chronic inflammation characteristic of inflammatory bowel diseases.

## Data Availability

The data in this work will be available when required.

## Abbreviations

The following abbreviations are used in this manuscript:

ATP	Adenosine triphosphate
CCL4	Chemokine (C-C motif) ligand 4
CCL5	Chemokine (C-C motif) ligand 5
CCL11	Chemokine (C-C motif) ligand 11
CCL17	Chemokine (C-C motif) ligand 17
CA	Crypt abscess
ME	Modified epithelial
EE	Eroded epithelium
FB	Fibrosis
MC	Mucin cells
GR	Granulomas
H2O2	Hydrogen peroxide
H-E	Hematoxylin and eosin
IBD	Inflammatory bowel disease
ICAM-1	Intercellular adhesion molecule 1
IL-1β	Interleukin-1 beta
IL-6	Interleukin-6
MDA	Malondialdehyde
MU	Micro ulcers
MV	Modify villi
NK	Natural killer cells
OM	Opening crypt
O3	Ozone
ppm	Parts per million
PP	Peyer’s patches
PLS	Plasmacytosis
PreHp-2	Prehaptoglobin-2
ROS	Reactive oxygen species
SDS-PAGE	Sodium Dodecyl Sulfate Polyacrylamide Gel Electrophoresis
SCFAs	Short-chain fatty acids
TBARS	Thiobarbituric acid reactive substances
TBA	Thiobarbituric acid
TJs	Tight junctions
TLRs	Toll-like receptors
VCAM-1	Vascular cell adhesion molecule 1
